# Cost, cost-consequence and cost-effectiveness evaluation of a practice change intervention to increase routine provision of antenatal care addressing maternal alcohol consumption

**DOI:** 10.1186/s13012-021-01180-6

**Published:** 2022-02-04

**Authors:** Zoe Szewczyk, Penny Reeves, Melanie Kingsland, Emma Doherty, Elizabeth Elliott, Luke Wolfenden, Tracey W. Tsang, Adrian Dunlop, Andrew Searles, John Wiggers

**Affiliations:** 1 Hunter Medical Research Institute, New Lambton Heights, New South Wales Australia; 2 School of Medicine and Public Health, The University of Newcastle, Callaghan, New South Wales Australia; 3 Hunter New England Population Health, Hunter New England Local Health District, Wallsend, New South Wales Australia; 4 School of Medicine, The University of Sydney, Camperdown, New South Wales Australia; 5 Sydney Children’s Hospital Network, Kids Research Institute, Westmead, New South Wales Australia; 6 Drug and Alcohol Clinical Services, Hunter New England Local Health District, Newcastle, New South Wales Australia

**Keywords:** economic evaluation, maternal and child, health service, alcohol drinking, implementation, cost

## Abstract

**Background:**

Implementation of antenatal clinical guideline recommendations for addressing maternal alcohol consumption is sub-optimal. There is a complete absence of evidence of the cost and cost-effectiveness of delivering practice change interventions addressing maternal alcohol consumption amongst women accessing maternity services. The study sought to determine the cost, cost-consequence and cost-effectiveness of developing and delivering a multi-strategy practice change intervention in three sectors of a health district in New South Wales, Australia.

**Methods:**

The trial-based economic analyses compared the costs and outcomes of the intervention to usual care over the 35-month period of the stepped-wedge trial. A health service provider perspective was selected to focus on the cost of delivering the practice change intervention, rather than the cost of delivering antenatal care itself. All costs are reported in Australian dollars ($AUD, 2019). Univariate and probabilistic sensitivity analyses assessed the effect of variation in intervention effect and costs.

**Results:**

The total cost of delivering the practice change intervention across all three sectors was $367,646, of which $40,871 (11%) were development costs and $326,774 (89%) were delivery costs. Labour costs comprised 70% of the total intervention delivery cost. A single practice change strategy, ‘educational meetings and educational materials’ contributed 65% of the delivery cost. Based on the trial’s primary efficacy outcome, the incremental cost effectiveness ratio was calculated to be $32,570 (95% CI: $32,566–$36,340) per percent increase in receipt of guideline recommended care. Based on the number of women attending the maternity services during the trial period, the average incremental cost per woman who received all guideline elements was $591 (Range: $329 - $940) . The average cost of the intervention per eligible clinician was $993 (Range: $640-$1928).

**Conclusion:**

The intervention was more effective than usual care, at an increased cost. Healthcare funders’ willingness to pay for this incremental effect is unknown. However, the strategic investment in systems change is expected to improve the efficiency of the practice change intervention over time. Given the positive trial findings, further research and monitoring is required to assess the sustainability of intervention effectiveness and whether economies of scale, or reduced costs of intervention delivery can be achieved without impact on outcomes.

**Trial registration:**

The trial was prospectively registered with the Australian and New Zealand Clinical Trials Registry, No. ACTRN12617000882325 (date registered: 16/06/2017).

**Supplementary Information:**

The online version contains supplementary material available at 10.1186/s13012-021-01180-6.

## Background

Alcohol consumption during pregnancy is associated with adverse obstetric and infant outcomes that can have lifelong social and economic consequences [1, 2]. Specifically, alcohol consumption during pregnancy increases the risk of miscarriage, still birth and Fetal Alcohol Spectrum Disorder (FASD) which is the most common preventable cause of intellectual impairment in the western world [2]. Despite this, the 2019 Australian National Drug Strategy Household survey reports that 55% of Australian women consumed any alcohol during pregnancy, and 14.5% continued to drink once they knew they were pregnant [3]. No safe level of alcohol exposure has been established and Australian national alcohol guidelines recommend women abstain from drinking alcohol whilst pregnant, trying to become pregnant, or breastfeeding [4].

Public maternity services are important settings for the provision of antenatal care to a large proportion of pregnant women [5, 6]. International [7] and Australian [8, 9] antenatal clinical practice guidelines recommend that during initial and subsequent antenatal appointments all pregnant women: have their alcohol consumption assessed; be advised that it is safest not to consume alcohol during pregnancy and of the potential risks of consumption; and be offered referral for additional alcohol treatment services if required [3]. Despite such guideline recommendations, assessment and care for antenatal alcohol consumption in public maternity services is sub-optimal [10, 11]. For example, in Canada approximately only half of surveyed health professionals reported providing advice to pregnant women regarding the consumption of alcohol [12]. In the United Kingdom two thirds of women reported receiving such advice from a midwife [13]. In a recent Australian survey less than two thirds of pregnant women reported that they received an assessment of their alcohol consumption and just over one third received advice and referral appropriate to their level of alcohol consumption at their initial antenatal visit [11]. Less than 10% of women received recommended care at subsequent antenatal visits [11]. Practice change strategies have been demonstrated to be effective in increasing the provision of evidence-based care in various clinical settings. Such strategies include educational meetings, local opinion leaders providing expert opinion, audit and feedback and electronic prompt and reminder systems [14–17]. No studies have reported the effectiveness of such strategies in improving the provision of care addressing maternal alcohol consumption by maternity services. Whilst effective, the delivery of these practice change strategies should be considered against their resource requirements [18].

Despite the increasing use of economic evaluation in health services research, its application to the assessment of the cost and cost effectiveness of practice change intervention strategies is limited [18] [19, 20]. A 2019 systematic review by Roberts et al. identified 30 studies that included implementation or improvement as part of an economic evaluation [20]. Of those, 14 were implementation studies and the most common focus was on implementation strategies of new care pathways or novel services [20]. Of these, seven included a cost-effectiveness analysis (CEA), of which two were conducted in the Australian setting, one was a web-based intervention [21] implementation designed to improve the management of minor head injury in emergency departments [22]. Of the 14 implementation studies identified in this review, only six included specific implementation costs, such as those associated with staff training and education, the impacts of new processes on patient and carer costs and the cost of developing new processes [20]. Another recent systematic review of economic evaluations and cost analyses of guideline implementation strategies identified 235 implementation studies, of which only 10% provided information about implementation costs, with none providing detailed cost information [23]. Furthermore, this review identified 63 studies (27%) that reported an economic evaluation, however, overall the methodological quality was poor and very few included conclusions on the effectiveness or efficiency of implementing the guideline into practice [23]. Similarly, a systematic review of economic evaluations of antenatal nutrition and alcohol interventions and their implementation identified 12 studies, ten addressing nutrition intervention effectiveness and two addressing alcohol interventions [24]. The review found that although the alcohol interventions were cost-effective or cost saving, the cost and cost-effectiveness of the intervention and its associated practice change interventions were not reported, and have not been reported previously [24]. Failure to identify, measure and value explicit costs associated with implementation risks underestimation of the investment required to change practice. This has been identified as a missed opportunity to develop evidence about the importance of fixed and recurring costs associated with practice change interventions [20].

The importance of economic evaluation in the context of implementation science and how these analyses can be most efficiently incorporated into decision-making process has been recognised [19]. To address this evidence gap, a trial-based economic evaluation was conducted of a practice change implementation intervention that aimed to improve delivery of guideline recommended antenatal care addressing alcohol consumption by women during pregnancy. The aims of the economic evaluation were to:

1. Identify, measure and value the cost of developing and delivering a multi-strategy practice change intervention;
2. Report the costs and consequences of a multi-strategy practice change intervention; and
3. Determine the cost-effectiveness of a multi-strategy practice change intervention in improving antenatal care provider adherence to antenatal care guidelines compared to usual care.

The secondary aims for the economic evaluation were to report each of the aims by sector.

## Methods

### The trial

The practice change intervention trial has been reported by Kingsland et al. [25]. In summary, a randomised stepped-wedge controlled trial of a maternal alcohol practice change intervention was conducted in maternity services in three sectors in the Hunter New England Local Health District (HNELHD), New South Wales, Australia. Combined, the sectors provide antenatal care for approximately 6,100 women annually, accounting for 70% of public hospital births in the district [25]. The sectors provided antenatal care to women in a major city (Sector One: 4300 births per annum) and two regional/rural areas (Sectors Two and Three: 1200 and 600 births per annum respectively). The participating maternity services provided antenatal care for women through hospital and community-based midwifery clinics; hospital medical clinics; midwifery continuity of care group practices; Aboriginal Maternal and Infant Health Services (AMIHS); and specialist services caring for women with complex pregnancies or social vulnerabilities. All antenatal care providers in these services were eligible to receive the implementation strategies, including midwifery and medical staff and Aboriginal Health Workers (AHWs).

Outcome data were collected over a 35 month period from seven-months prior to commencement of the practice change intervention in the first sector to seven-months following completion of the intervention in the third sector [11]. Stepped delivery of the seven-month intervention period in each of the three sectors occurred in a random order at seven-month intervals [25]. All antenatal care providers who worked in the participating maternity services were eligible to receive the practice change intervention. The trial primary outcome measures were the proportion of antenatal appointments at ‘booking in’ (initial antenatal , 27–28 weeks gestation and 35–36 weeks gestation for which women report [1] being assessed for alcohol consumption, [2] being provided with brief advice related to alcohol consumption during pregnancy, [3] receiving relevant care for addressing alcohol consumption during pregnancy, and [4] being assessed for alcohol consumption and receiving relevant care [25].

### Guideline recommended model of antenatal care

An evidence- and guideline-based model of antenatal care was developed to address alcohol consumption by pregnant women [25]. The model of care involved health care provider assessment of a woman’s alcohol risk status using the AUDIT-C tool at the initial antenatal visit, and at follow up antenatal appointments at 27-29 weeks and 35-37 weeks gestation. The model of care also required all pregnant women were to be provided with brief advice indicating it is safest not to consume alcohol during pregnancy and of the risks associated with alcohol consumption. Women who were assessed as being at ‘medium risk’ of harm (AUDIT-C score: 3-4) were to be offered a referral to the NSW Get Healthy in Pregnancy Service, a free government provided evidence-based telephone coaching service for Aboriginal women, or to a face-to-face counselling service where available. Women who were assessed as being at ‘high risk’ of harm (AUDIT C score: 5+) were referred to Hunter New England Drug and Alcohol Clinical Services for further assessment and follow-up.

### Practice change intervention

A multi-strategy practice change intervention to support the provision of the antenatal model of antenatal care was co-designed with input from health service stakeholders (e.g. senior maternity service staff, drug and alcohol service staff) and Aboriginal health organisations and women, and was guided by an implementation framework [26–29]. The intervention involved seven evidence-based practice change strategies: leadership and management; local clinical practice guidelines; electronic prompt and reminder system; local clinical/academic opinion leaders (change champions); educational meetings and educational materials; academic detailing (including audit and feedback); and monitoring and accountability for the performance of the delivery of health care [25]. Refer to Table 1.

### Usual care

Prior to delivery of the practice change intervention in each of the three sectors, usual antenatal care addressing maternal alcohol consumption during pregnancy was provided [11]. An observational study was conducted in 2017-2018 to examine pregnant women’s reported receipt of guideline recommended care addressing alcohol consumption during pregnancy [11]. The study found that although assessment and care for maternal alcohol consumption is highly acceptable to pregnant women, receipt of such care in public antenatal services is suboptimal and inconsistent [11].

### Economic evaluation

Details of the approach to conducting the economic evaluation have been reported in the economic evaluation protocol [30]. A trial-based economic evaluation was conducted to assess cost, cost-consequence and cost-effectiveness of the development and delivery of the intervention. The analysis was conducted from a healthcare provider perspective and was based on opportunity cost. The perspective was chosen as ongoing investment in the intervention, if translated into routine practice, would fall on public health services [30]. Costs incurred in 2017 and 2018 were adjusted for inflation using annual consumer price index [31]. All costs are reported in 2019 Australian dollars ($AUD).

The economic evaluation was conducted and reported in accordance with the Consolidated Health Economic Evaluation Reporting Standards (CHEERS) publication guidelines and good reporting practices [32]. Adherence to the CHEERS checklist is available in Supplementary Table 1.

### Identification and measurement of outcomes

The economic evaluation was based on the trial primary outcome: the proportion of women at the initial, 27-28 weeks gestation and 35–36 weeks gestation antenatal visits who reported being assessed for alcohol consumption using the Alcohol Use Disorders Identification Test (AUDIT-C) tool, and who received the recommended elements of care (advice and referral) appropriate to their level of risk, as determined by AUDIT-C risk categories for pregnancy [30]. The trial primary outcome and the economic cost data were combined in an incremental cost-effectiveness ratio (ICER) calculated as the incremental cost per percentage increase in self-reported receipt of all guideline elements. ICERS by sector were reported as secondary outcomes.

### Identification, measurement and valuation of practice change costs

At present, there are no guidelines for costing practice change interventions that aim to improve the implementation of guideline recommendations in health care settings [33, 34]. A recent pragmatic method for costing implementation strategies using time-driven activity based costing has been proposed by Cidav et al [33]. Time-driven activity-based costing is a micro-costing method widely used in business settings, which delivers detailed, accurate and transparent information on activity costs designed to inform quality assurance processes and decision making [33, 35, 36]. The costing method involves three parameters; (i) frequency of the activity, (ii) time required to perform one single event of the activity, and (iii) per-hour price of the resources used to perform the activity [33]. The detailed information collected using this approach provides a direct link between the implementation inputs (resources utilized) and implementation outcomes [33]. Cidav’s methods were used to inform the identification, measurement and valuation of implementation intervention data in the economic evaluation [33].

Intervention costs were prospectively identified and measured using a time-driven activity based cost-capture tool [30]. The cost-capture tool was developed in Microsoft Excel (2013) and allowed researchers to document the activity and materials consumed at different phases of the trial (development and delivery) and for all relevant stakeholders [30]. The cost-capture tool included the following resource use categories: [1] Labour [2]; Materials; and [3] Miscellaneous costs. Table 2 presents the approach to the valuation of unit costs for each item. Researcher officers involved in the trial delivery completed the cost-capture tool at the time of the cost being incurred throughout the trial duration. Labour, materials, and other implementation costs were captured, with the cost-capture tool built to allow expenses to be allocated to pre-coded cost categories and to one or more pre-coded implementation strategies. Following the reporting convention established in the CHEERs checklist, development and research costs are deemed ‘start-up’ costs [1]. We excluded development costs in this analysis as they represent the investment made by the research team to formulate the intervention components, and are not representative of the resource use required in ‘steady state’ operations. However, we separately reported the calculated value of the development costs to inform the upfront investment required to develop this intervention, where no similar model of care exists. Research related costs together with intervention development costs were excluded from the cost-consequence and cost-effectiveness analysis to achieve a focus on the costs and efficiency of the practice change intervention alone.

Cost data were treated as counts of resource use, weighted by unit costs. The cost for each sector was determined by summing the intervention delivery costs relevant to and coded for that sector. A cost per practice change strategy is reported to demonstrate the investment required for each of the seven strategies and to inform future intervention scale up and sustainability. The intervention was wholly additional to usual care, that is, no usual practice activity was displaced as a result of the intervention. Costs to providers, patients and private care providers (including opportunity costs) were not assessed.

### Cost-consequence (CCA) and cost-effective analyses (CEA)

The results of the CCA are presented as the total cost of delivering the intervention alongside the range of outcomes reflected in the primary and secondary trial outcomes (consequences). The cost per eligible clinical provider is presented to demonstrate the cost and consequence of the intervention for those intended to provide the evidence-based model of care. Eligible clinical providers were all clinicians within the participating maternity services who provided antenatal care during the intervention period. Eligible clinical providers included midwifery and medical staff as well as Aboriginal Health Workers. It excluded clinicians who were not the primary providers of antenatal care (e.g. dietitians, diabetes educators and drug and alcohol clinicians). Eligible clinical providers were identified through rostering and payroll systems obtained from the participating maternity services. Modelled extrapolation of the self-report survey data was conducted to estimate the proportion of all women attending the participating maternity services who received all guideline elements during the intervention follow-up period. This extrapolation enabled estimation of the incremental cost per woman who received all guideline elements, in each sector. The average cost per woman was calculated as the cost of the practice change intervention, divided by the total number of women who are anticipated to have received all guideline elements based on the sample of women surveyed.

The trial-based CEA aligned the cost of the intervention against self-reported receipt of all guideline elements of antenatal care. The trial outcomes, reported as odds ratios, were converted to risk differences for inclusion in the CEA, e.g. the risk difference for the primary outcome refers to the percentage point (proportion) increase in the self-reported receipt of all guideline elements of antenatal care. This information was used to generate an ICER.

### Uncertainty, sensitivity and sub-group analyses of ICERs

ICERs were calculated by sector to enable reporting of the variation in costs and effect sizes between the three sectors. One-way probabilistic sensitivity analyses assessed the effect of variation in the magnitude of treatment effect using the lower and upper confidence interval limits and variation in costs of intervention components using the lower and upper bounds of staff salaries. Non-parametric bootstrapping was undertaken to derive uncertainty intervals around the estimates for total cost and cost-effectiveness. Results from the sensitivity analyses were visually presented on a cost-effectiveness plane where the joint distribution of incremental costs were plotted against the incremental change in effect size between baseline and follow-up. Refer to Figs. 2 and 3.

## Results

### Cost

The resources invested to increase compliance with the guideline-based model of care were calculated as being wholly incremental to usual practice. The total cost of the practice change intervention across all three sectors was $367,646 of which $40,871 (11%) were development costs and $326,774 (89%) ($AUD, 2019) were intervention delivery costs. Of the intervention delivery costs, Sector one cost $133,188 (41%) of the practice change intervention costs. Sectors two and three incurred similar proportions of the practice change intervention cost, 30% and 29% respectively. The cost of labour was the main cost driver for the intervention, comprising 70% of the total intervention costs. Refer to Table 3.

**Fig. 1:**
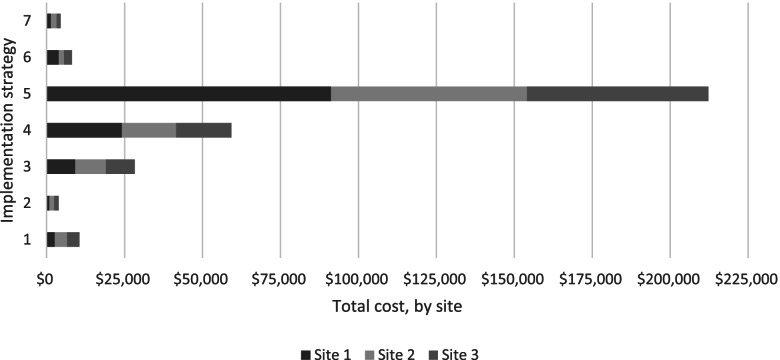
Cost ($AUD, 2019) per practice change strategy, by sector. Practice change strategies: 1) Leadership/managerial supervision; 2) Local clinical practice guidelines; 3) Electronic prompt and reminder system; 4) Local opinion leaders/champions; 5) Educational meetings and educational materials; 6) Academic detailing, including audit and feedback; 7) Monitoring and accountability for the performance of the delivery of healthcare

Of the seven practice change intervention strategies ‘educational meetings and educational materials’ comprised 65% of the total intervention cost, followed by ‘local opinion leaders/champions’ with 18% of the intervention costs. The salary of the clinician midwife educator (CME) was the main cost driver for the ‘educational meetings and educational materials’ strategy. A CME was employed in each sector for the duration of the 7-month intervention. The CME for Sector one was employed at 1.0 full-time equivalent (FTE), the CME for Sector two at 0.6 FTE and Sector three at 0.4 FTE, relative to the number of births in each Sector. The remaining five intervention strategies each incurred between 1% and 8% of the total intervention costs. Refer to Fig. 1.

### Cost-consequence and cost-effectiveness analyses

Significant intervention effects were found for receipt of all guideline elements (risk difference 9.33; 95% CI 7.67–10.98; *p* = <0.001). The increase in receipt of all guideline elements was seen across all three sectors. The average cost of delivering the practice change intervention per eligible clinician was $993 (Range: $640-$1928). Based on the average number of women in the service per month over the trial follow-up period, the extrapolated average cost per woman who received all guideline elements was calculated to be $591 (Range: $329 - $940). Variation in the average cost per woman was associated with variation in the average number of women through each service, per month between sectors. Sector one had a total of 6862 women through the antenatal service during the 21-month follow-up period, an average of 58 women per month reporting receipt of all guideline elements for the duration of the intervention. In comparison, Sector two had an average of 29 women per month reporting receipt of all guideline elements for a 14-month time period and Sector three had an average of 14 women per month for the seven-month time period. Refer to Table 4.

The calculated ICER per percentage point increase in self-reported receipt of all guideline elements was $32,570 (95% CI: $32,566–$36,340). The incremental cost per percent increase in women receiving all guideline elements ranged from $15,951 (95% CI: $13,109–$20,365) in Sector one and $5,618 (95% CI: $4,261–$8,25) in Sector two. Refer to Table 5.

### Sensitivity analysis

The ICER in the sensitivity analysis was $35,024 (95% CI; $29,761 - $42,604), indicating that the ICER was sensitive to variation in labour costs Table 5.

**Fig. 2:**
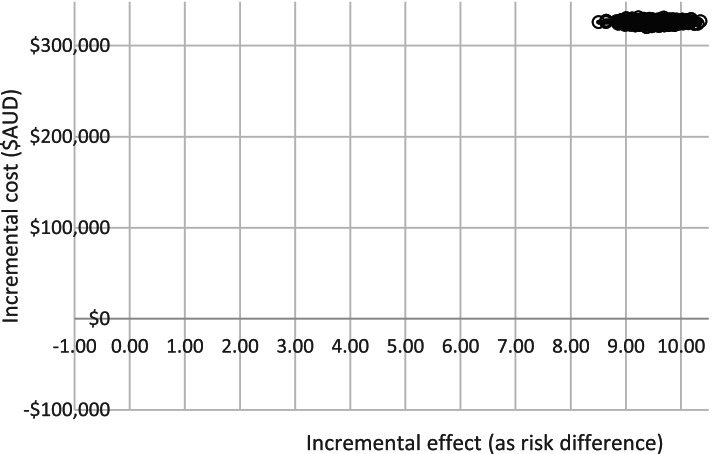
Cost effectiveness plane

**Fig. 3:**
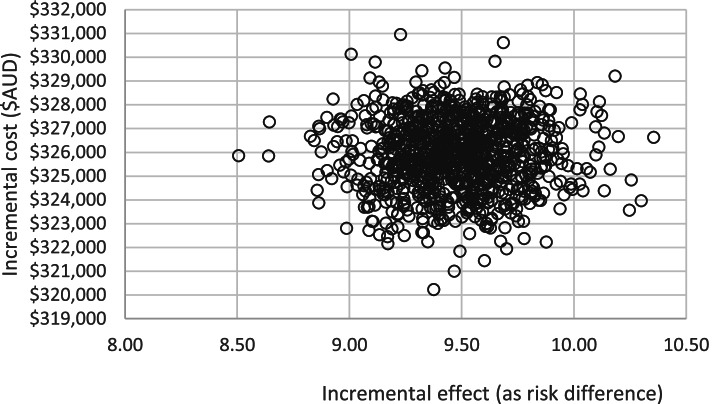
Cost-effectiveness plane area of interest

Figures 2 and 3 present the joint distribution of incremental intervention cost and incremental effectiveness. All replications show a positive incremental benefit of the intervention over usual care, at increased cost. Figure 2 is a focussed view demonstrating limited variation of ICER pairs.

## Discussion

This economic evaluation outlined the costs, consequences, and cost-effectiveness associated with the first published randomised controlled trial to examine the effectiveness and efficiency of delivering a practice change intervention to improve the implementation of guideline recommended care for maternal alcohol consumption. The intervention was effective at improving reported receipt of all guideline elements of care. The incremental cost of intervention delivery was calculated to be $326,774. Labour (staff time) was the main cost driver and variation in delivery costs across sectors was associated with design differences in labour time and travel costs for each sector.

The average cost over the trial period of delivering the practice change intervention per eligible clinician was $993, and $591 per woman who received all guideline elements. The cost of delivering clinician training was upfront and is not expected to require additional investment beyond the trial horizon. Clinician training modules were developed during the intervention and made permanently available online for staff to access as part of their ongoing clinician training. Further, in an effort to sustain the intervention effect size, implementation strategies were deliberately designed to affect permanent and scalable change within the health system, e.g. the inclusion of specific modules into the state-based e-maternity patient record management system, and performance measures were embedded into the health system. Therefore, these implementation strategies are expected to sustain the effect size post-intervention. As such, the average cost per woman is expected to reduce markedly with continued delivery of this new model of care over time (e.g. beyond the trial time horizon), with increased women receiving antenatal care and with scaling up to involve other maternity services/clinicians. Similarly, with respect to potential ongoing costs (beyond the trial time horizon) associated with this intervention and maintaining the intervention effect size beyond the trial time horizon.

Direct comparison between the results of this and previously reported studies of the cost and cost effectiveness of practice change interventions is not possible given the significant differences in the design and methods of the various studies. Broad comparisons with studies included in previous systematic reviews were also limited as the costs associated with the implementation of the practice change strategies is not often considered [20, 24, 37]. The review by Roberts et al. [20] identified one modelled CEA of a universal alcohol screening and brief intervention program in primary care in England [38]. This analysis compared the health and social care costs verses health benefits and found screening patients for alcohol consumption upon registration with a family doctor would steadily capture up to 96% of the population over a 10-year programme [38]. This study showed that alcohol screening and the provision of brief advice, led by practice nurses, provided cost savings to the health care system of £120m over 30 years [38]. Similar to the methods and findings of the present study, the cost of the intervention was estimated using activity costs and identified that resourcing needs for this intervention would be highest in the early years of the program due to the volume of new patients being screened, and would decrease over time [38]. This study did not include the cost of implementing this model of care into routine practice or addressing barriers to care delivery by relevant health service providers. In comparison, the practice change strategies used in the current study were designed to be embedded into the health care system to maintain the new model of care beyond the intervention delivery and follow-up period. As such, it is plausible that the demonstrated change in practice could be sustained beyond the trial time horizon and the average cost per woman screened would decrease as more women attend the antenatal service. Future studies could include longer intervention follow-up periods to capture the number of women receiving care over a longer time period, as well as the health and societal benefits for mothers and their infants. Further research is also required to determine the cost of delivering the intervention at scale and whether economies of scale can be achieved in its delivery.

Implementation costs are recognised as an understudied aspect of implementation science [39, 40]. Saldana et al. proposed that one of the reasons implementation costs are not routinely examined is the lack of standard measurement [39]. More recently, Cidav et al. proposed a pragmatic approach to systematically estimating detailed resource use and costs of implementation strategies that combine time-driven activity-based costing with a leading implementation science framework [41] to guide specification and reporting of implementation strategies [33]. One of the key strengths of this study was the use of this time-driven activity based micro-costing to map implementation processes with actions, actors and strategies. This method provides transparent, granular cost estimation and allowed for a cost comparison of the different implementation strategies. It has been proposed that there could be value to using standardised methods for estimating implementation costs as it could allow decision makers responsible for determining the viability and feasibility of adopting new practices to benefit from the ability to generalise across settings [39, 42]. The transparent reporting of activity costs using a published time-driven activity-based costing method is intended to inform researchers and decision makers how specific components of an implementation intervention influence the total cost.

Another strength of this economic evaluation is the identification, measurement and valuation of development costs. That is, once the practice change intervention has been delivered, there are non-recoverable intervention components that remain within each sector. For example, the cost of developing and producing local clinical guidelines, the cost of developing educational materials and the cost of developing site-specific systems level monitoring and accountability measures. Saldana et al. argue development costs are an important consideration in decision making as policy makers must decide ex ante whether to invest in a new model of care and unrecoverable costs must be considered amongst the future benefits [39]. Transparent reporting of costs associated with the practice change intervention is intended to inform decision makers of when, during the practice change process, different costs and benefits can be expected [19].

It was not possible to calculate an ICER for the cost per additional service user (pregnant woman) who received all guideline elements of care. The study was designed and powered to measure effectiveness at the health sector level, thus precluding this ICER calculation. Given this limitation, the average cost per additional service user (pregnant woman) exposed to the recommended treatment was calculated. Another, limitation of the study was that the ICER was calculated on the assumption that the intervention was wholly additional to usual care, that is, no usual practice activity was displaced as a result of the intervention. Identifying, measuring and valuing the cost of usual care was beyond the scope of the trial and hence a limitation of the analysis. The trial time horizon was 35-months, and as such only upfront and short-term costs to health service providers were included. Similarly, the potential impact of increased referrals to drug and alcohol services and the longer-term benefits of alcohol-harm reduction to society at large, was not captured in this trial and is a noted limitation. Future economic evaluations should endeavour to include these components. The range of costs associated with sustaining changes in provider behaviour and maternal alcohol consumption is complex [2], and beyond the scope of the analyses.

## Conclusion

The practice change intervention was effective at improving women’s reported receipt of all guideline elements of care. The incremental cost of delivering the intervention was calculated to be $326,774. To our knowledge, no similar studies have been published in the literature. The economic evaluation provides information for decision and policy makers regarding the cost, cost-consequence and cost-effectiveness of delivering a practice change intervention to support the introduction of a model of care for addressing alcohol consumption by pregnant women. Given the positive trial findings, further research is required to assess sustainability and determine the cost of delivering the intervention at scale and whether economies of scale can be achieved.

### Supplementary Information

**Additional file 1.**

## Data Availability

The datasets used and/or analysed during the current study are available from the corresponding author on reasonable request.

## Appendices

**Table 1:** Implementation Strategy Summary

Intervention component	Component details	Resource use details	Data collection method for costing
**Leadership and management**	• Monthly meetings were held with management from antenatal services to elicit support. • Service managers distributed resources to staff and attended training sessions. • Performance measures related to the provision of the model of care were monitored and reported on.	**Labour time:** • Health district implementation support officer and manager. • Health service antenatal clinical staff and management.	• Resource use capture template
**Local clinical practice guidelines**	• A service level guideline and procedure document detailed the model of care, including assessment, brief advice and referral pathways. • The document was uploaded onto the health service’s policy directory, disseminated by managers to all staff via email and hard copies were placed in staff common areas.	**Materials:** • Guideline and procedure document development and provision. **Miscellaneous:** • Electronic dissemination. **Labour time:** • Health district implementation support officer and manager. • Health service antenatal clinical staff and management.	• Resource use capture template
**Electronic prompt and reminder system**	• Existing point-of-care and medical record systems used by maternity clinicians were modified to electronically prompt use of the AUDIT-C alcohol screening tool. • Brief advice scripts were displayed on the point-of-care system based on the woman’s AUDIT-C risk score and prompts and tools for referral to appropriate services.	**Materials:** • Computer-based intervention component. **Labour time:** • Health district implementation support officer and manager. • Health service antenatal clinical staff and management.	• Resource use capture template
**Local opinion leaders/champions**	• Project-specific Clinical Midwife Educators were appointed to support staff to uptake the model of care and provide support at a one-on-one, team and service level. • Additional local antenatal clinical leaders were engaged to provide encouragement and demonstrate required behaviours as required.	**Labour time:** • Health district implementation support officer. • Clinical midwife educator (CME) change champion.	• Resource use capture template
**Educational meetings and materials**	• Training was provided to all antenatal service clinicians via a 30-minute online training module and face-to-face sessions. Clinical Midwife Educators facilitated clinicians completing the online training and coordinate face-to-face training sessions. This included lecture style sessions, interactive, case-study based sessions and one-on-one sessions. • Clinicians were provided with written resources (hardcopy and electronic) to support the model of care, including standard drink measure charts and point-of care written prompts/reminders (e.g. stickers in charts).	**Labour time:** • Health district implementation support officer. • CME change champion. • Health service clinical staff. • Expert clinicians. **Materials:** • Educational tools and resources	• Resource use capture template • REDCap database
**Academic detailing**	• Data from both medical records and telephone surveys conducted with women who attended the antenatal services were used to provide feedback on adherence to the agreed model of care. • The Clinical Midwife Educators visited service teams in their antenatal clinics to provide feedback data and developed action plans to improve adherence.	**Labour time:** • Project support officer. • CME change champion. • Clinical service staff time.	• Resource use capture template • REDCap database
**Monitoring and accountability**	• Antenatal service managers reported, interpreted and monitored performance measures for the model of care. • These results were disseminated to antenatal service staff through team meetings, emails and other usual communication mechanisms. • Performance measures were built into the existing monitoring and accountability frameworks for antenatal services.	**Labour time:** • Health district implementation support officer. • Health service antenatal clinical staff and management. **Miscellaneous:** • Electronic dissemination.	• Resource use capture template

**Table 2:** Approach to valuation of resources, by cost category

Item	Description	Approach to valuation
Labour time	Health service labour time incurred during intervention development and implementation	Staff time was recorded in minutes and NSW Health staff grade was recorded in trial management logs and cost-capture templates. Labour time was valued using NSW Health Award 2019*
	Non-health service labour time incurred during intervention development and implementation	Staff time was recorded in minutes and job title was recorded in trial management logs and cost-capture templates. Labour time was valued using Fair Work Australia Award Wages or University of Newcastle Academic Staff and Teachers or Professional Staff enterprise agreement*
Materials	Material items used during intervention development and implementation. For example, changes to electronic medical records system, printed resources, and stickers	Purchase receipts and trial management logs were used to value material items.
Miscellaneous	Included catering for training sessions and staff travel allowance or use of fleet vehicle	Purchase receipts and trial management logs were used to value all miscellaneous items.

*Labour time was costed at 1.3 to account for additional overhead costs (on-costs) associated with employment

**Table 3:** Total intervention cost disaggregated by sector, resource use category and practice change strategy

	Total	Sector 1	Sector 2	Sector 3
**Total intervention costs**				
Total intervention development and practice change cost	$367,646	$154,927	$112,985	$99,733
Practice change intervention development cost	$40,871	$21,739	$13,883	$5,250
Practice change intervention delivery cost	$326,774	$133,188	$99,103	$94,483
**Costs: by resource use category**				
Labour cost	$229,566	$102,468	$64,692	$62,406
Material cost	$75,424	$25,338	$25,043	$25,043
Miscellaneous cost	$21,785	$5,383	$9,368	$7,034
**Cost by strategy**				
Leadership/managerial supervision	$10,528	$2,599	$3,940	$3,990
Local clinical practice guidelines	$3,875	$876	$1,484	$1,515
Electronic prompt and reminder system	$28,286	$9,171	$9,830	$9,285
Local opinion leaders/champions	$59,255	$24,126	$17,366	$17,763
Educational meetings and educational materials	$212,260	$91,224	$62,888	$58,148
Academic detailing, including audit and feedback	$8,100	$3,834	$1,715	$2,551
Monitoring and accountability for the performance of the delivery of healthcare	$4,471	$1,358	$1,881	$1,232

**Table 4:** Cost-consequence results (baseline to follow-up)

	Total	Sector 1	Sector 2	Sector 3
**Cost**				
Practice change intervention cost	$326,774	$133,188	$99,103	$94,483
**Consequences**				
**Women reporting receipt of all guideline elements at baseline**				
Mean (%) across all sites	13%	**-**	**-**	**-**
**Women reporting receipt of all guideline elements**				
Risk difference, post vs baseline (95% CI)*	9.33	8.35	17.64	12. 21
	(p <0.001)	(p <0.001)	(p <0.001)	(p <0.001)
	7.67–10.98	6.54–10.16	12.01–23.26	4.47–19.95
**Eligible antenatal providers who received the intervention**				
Total	329	208	72	49
Proportion	100%	63%	22%	15%
Average cost per eligible provider	$993	$640	$1,376	$1,928
**Women reporting receipt of all guideline elements of care**				
Time period (follow-up data collection)	Sept 2018 – May 2019	Sept 2018 – May 2020	Apr 2019 – May 2020	Nov 2019 – May 2020
Number of months	21	21	14	7
Proportion women who reported receiving all guideline elements	19%	18%	31%	27%
Total number of women who received antenatal care	8539	6862	1302	375
Total number of women who received all guideline elements	1658	1215	402	101
Average cost per woman	$591	$329	$493	$940
Average number of women who received all guideline elements, per month	79	58	29	14

* The primary outcome was reported as an odds ratio in the primary outcome’s manuscript. For the purpose of economic evaluation, it was converted to risk difference for inclusion in an ICER

**Table 5:** Incremental cost-effectiveness ratios for the primary and secondary outcomes

	ICER	95% CI	
**ICER for women reporting receipt of all guideline elements**			
Total	$32,570	$32,566 - $36,340	
**ICER per sector**			
Sector 1	$15,951	$13,109	$20,365
Sector 2	$5,618	$4,261	$8,252
Sector 3	$7,738	$4,736	$21,137

## Abbreviations

AUD: Australian dollars
AUDIT-C: Alcohol Use Disorders Identification Test
BIA: Budget impact assessment
CEA: Cost-effectiveness evaluation
DCEA: Distributional cost-effectiveness analysis
ICER: Incremental cost-effectiveness ratio
